# A Case Report of Amyand’s Hernia in a Previously Mesh-Repaired Groin: Surgical Decision-Making in a Contaminated Field

**DOI:** 10.7759/cureus.108852

**Published:** 2026-05-14

**Authors:** Giasar Chasan, Dimitrios Babalis, Sempaetin Gkasil, Lana Kalaitsidou, Vasiliki Tzelepi, Ilker Memet, Evanthia Tsalkidou

**Affiliations:** 1 Surgery Department, General Hospital of Komotini, Komotini, GRC; 2 Emergency Department, General Hospital of Komotini, Komotini, GRC; 3 Anesthesiology Department, General Hospital of Komotini, Komotini, GRC

**Keywords:** amyand’s hernia, appendectomy, appendicitis, case report, contaminated field, emergency surgery, inguinal hernia, mesh repair, recurrent hernia, surgical decision-making

## Abstract

Amyand’s hernia is a rare clinical entity defined by the presence of the vermiform appendix within an inguinal hernia sac, and its management remains controversial, particularly in the presence of contamination and prior mesh repair. We report the case of a 74-year-old man presenting with acute right inguinal pain suggestive of a strangulated recurrent inguinal hernia. Intraoperatively, a gangrenous appendix was identified within the hernia sac with purulent contamination. Appendectomy and peritoneal lavage were performed via a combined inguinal and McBurney approach. Due to contamination, no new mesh was placed, while the previously implanted mesh was preserved. Microbiological analysis revealed *Escherichia coli* sensitive to the administered antibiotic regimen, and the patient received piperacillin/tazobactam and metronidazole. The postoperative course was uneventful, and at one-month follow-up, the patient remained asymptomatic with no evidence of recurrence. This case highlights the importance of individualized surgical decision-making in complex contaminated hernia cases, particularly regarding the management of previously implanted mesh.

## Introduction

Amyand’s hernia is defined as the presence of the vermiform appendix within an inguinal hernia sac and accounts for approximately 1% of inguinal hernias [1,2]. Acute appendicitis within the hernia sac is even rarer, reported in approximately 0.1% of cases [2].

Preoperative diagnosis is challenging due to the nonspecific clinical presentation, and most cases are identified intraoperatively [3]. Management is largely determined by intraoperative findings, particularly the condition of the appendix and the presence of contamination. The use of prosthetic mesh in such cases remains controversial [4].

This case report has been reported in line with the Surgical CAse REport (SCARE) 2025 criteria. This case is important to document because it highlights the complex intraoperative decision-making required in contaminated Amyand’s hernia with previously implanted mesh, a clinical scenario for which limited evidence and surgical guidance currently exist.

## Case presentation

In March 2026, a 74-year-old man presented to the emergency department with acute right inguinal pain of approximately 24-hour duration. Clinical examination revealed a tender, non-reducible right inguinal mass, raising the suspicion of a strangulated recurrent inguinal hernia.

The patient had a history of bilateral inguinal hernia repair with mesh and no other significant comorbidities apart from controlled hypertension. No preoperative imaging was performed due to the urgent clinical presentation.

Laboratory evaluation revealed leukocytosis with neutrophilia and elevated inflammatory markers (Table 1).

**Table 1:** Laboratory findings on admission

Parameter	Patient Value	Reference Range
White blood cells (WBC)	Elevated	4,000-10,000/μL
Neutrophils	Elevated	40%-70%
C-reactive protein (CRP)	1.4 → 39 mg/L	<5 mg/L

**Figure 1:**
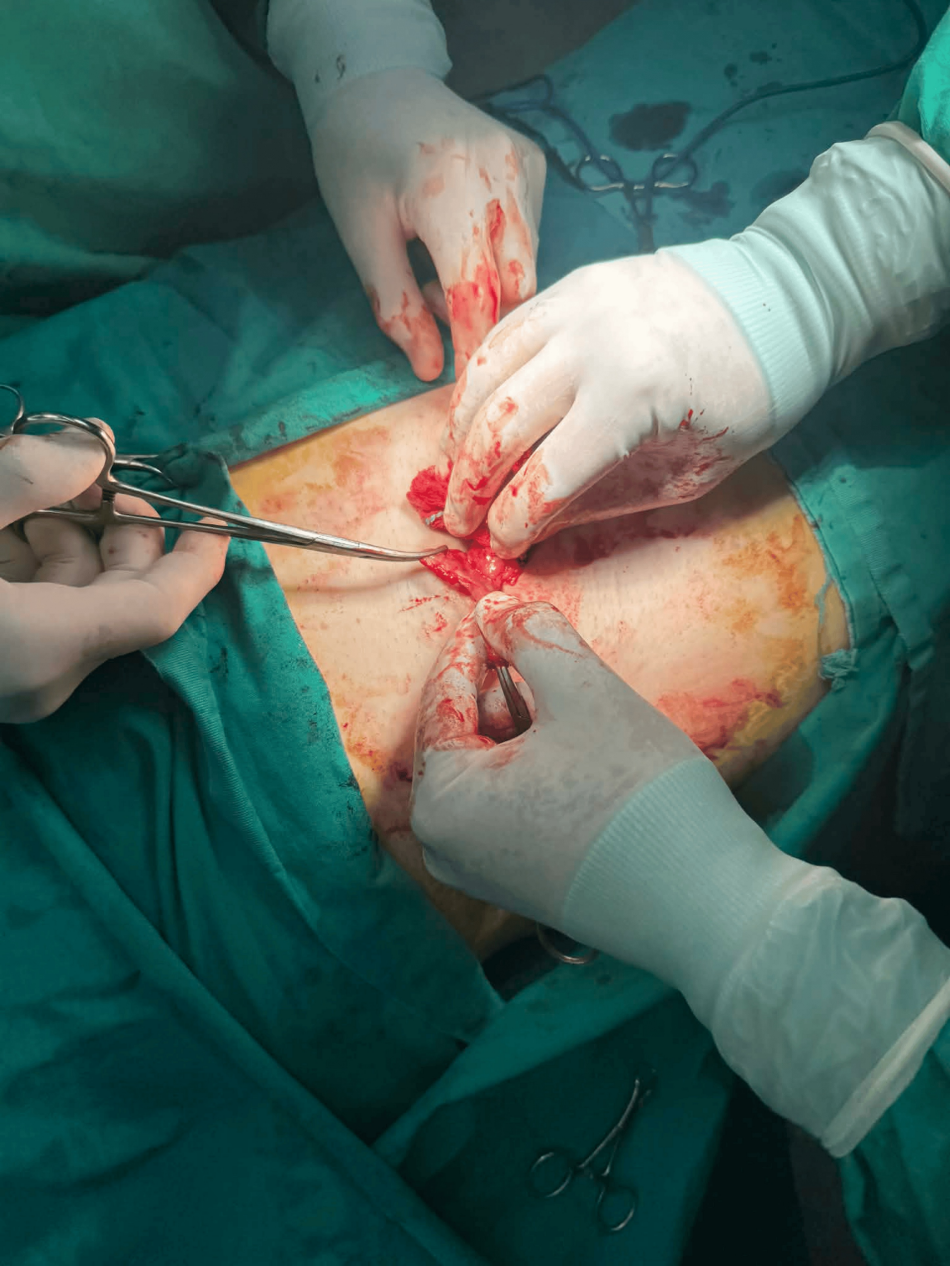
Mobilization and exteriorization of the appendix through the inguinal incision Intraoperative image showing the careful mobilization and exteriorization of the appendix through the inguinal approach prior to appendectomy

Intraoperatively, a gangrenous appendix was identified within the hernia sac (Figure 1).

**Figure 2:**
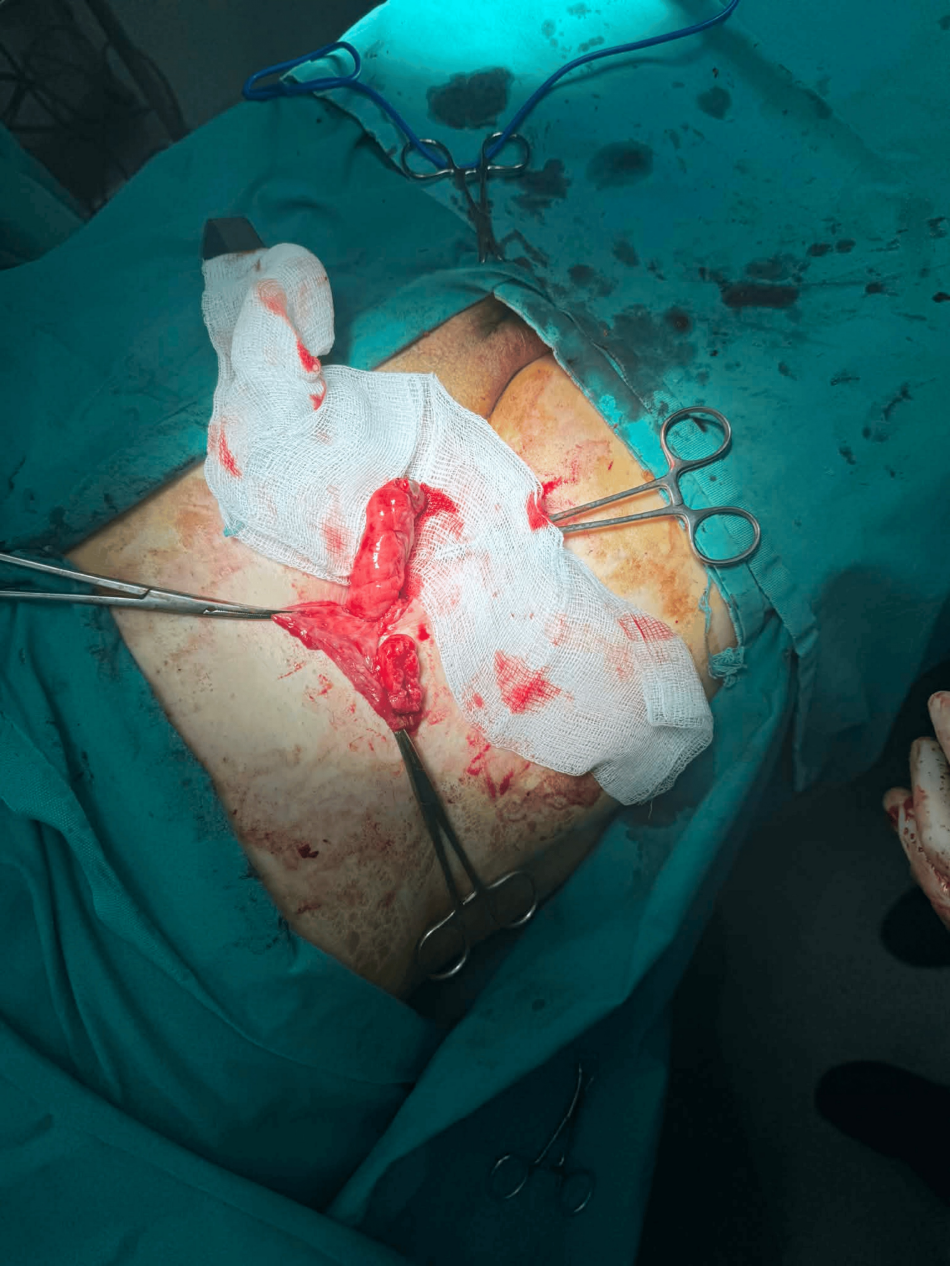
Appendectomy and exteriorization of the appendix through the inguinal incision Intraoperative image demonstrating appendectomy following the mobilization and exteriorization of the appendix through the inguinal approach

The appendix was carefully mobilized and resected (Figure 2).

**Figure 3:**
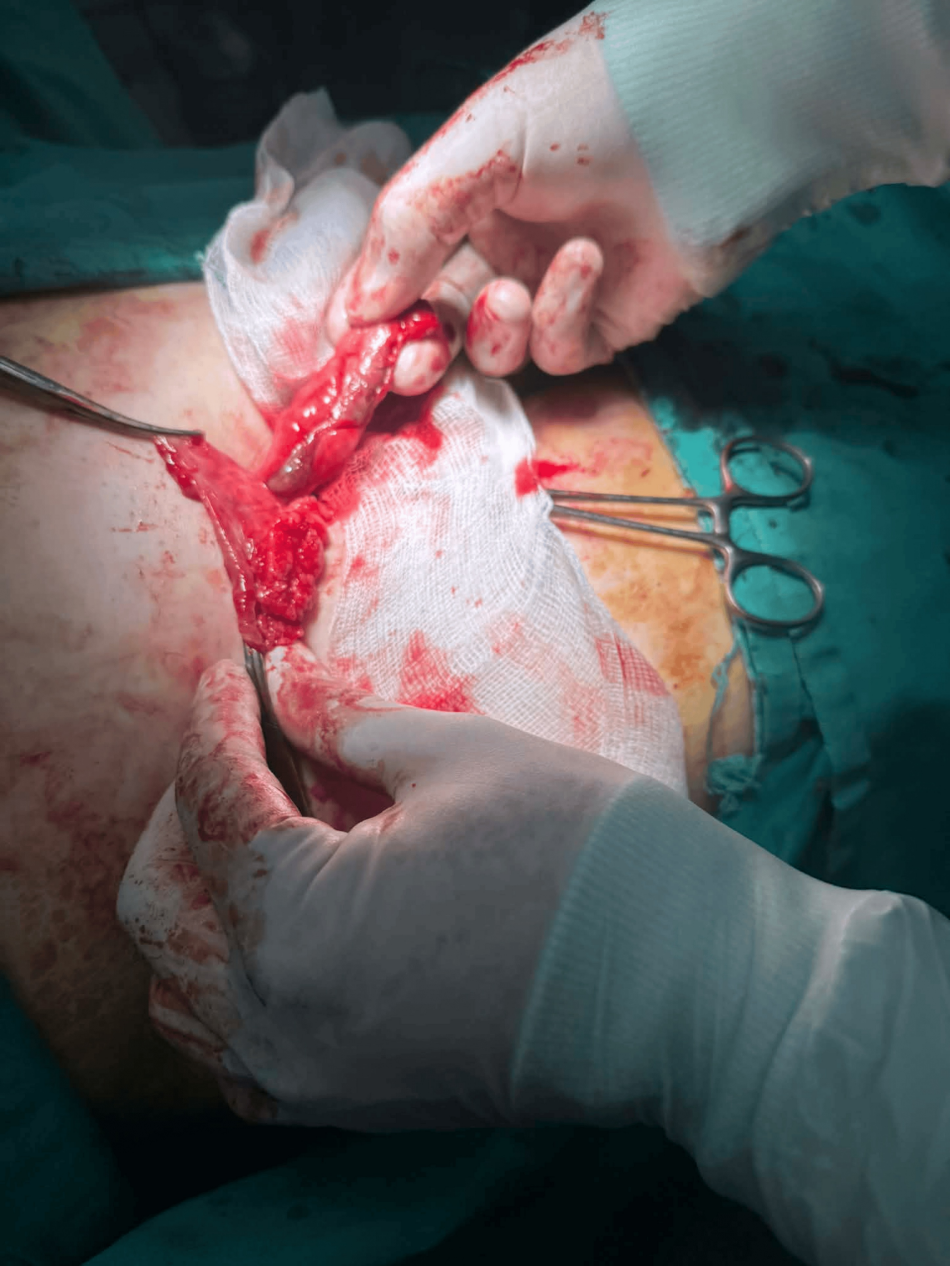
Intraoperative view demonstrating purulent contamination within the surgical field Purulent fluid identified during exploration, confirming the local contamination of the operative field

Purulent contamination was observed intraoperatively (Figure 3).

**Figure 4:**
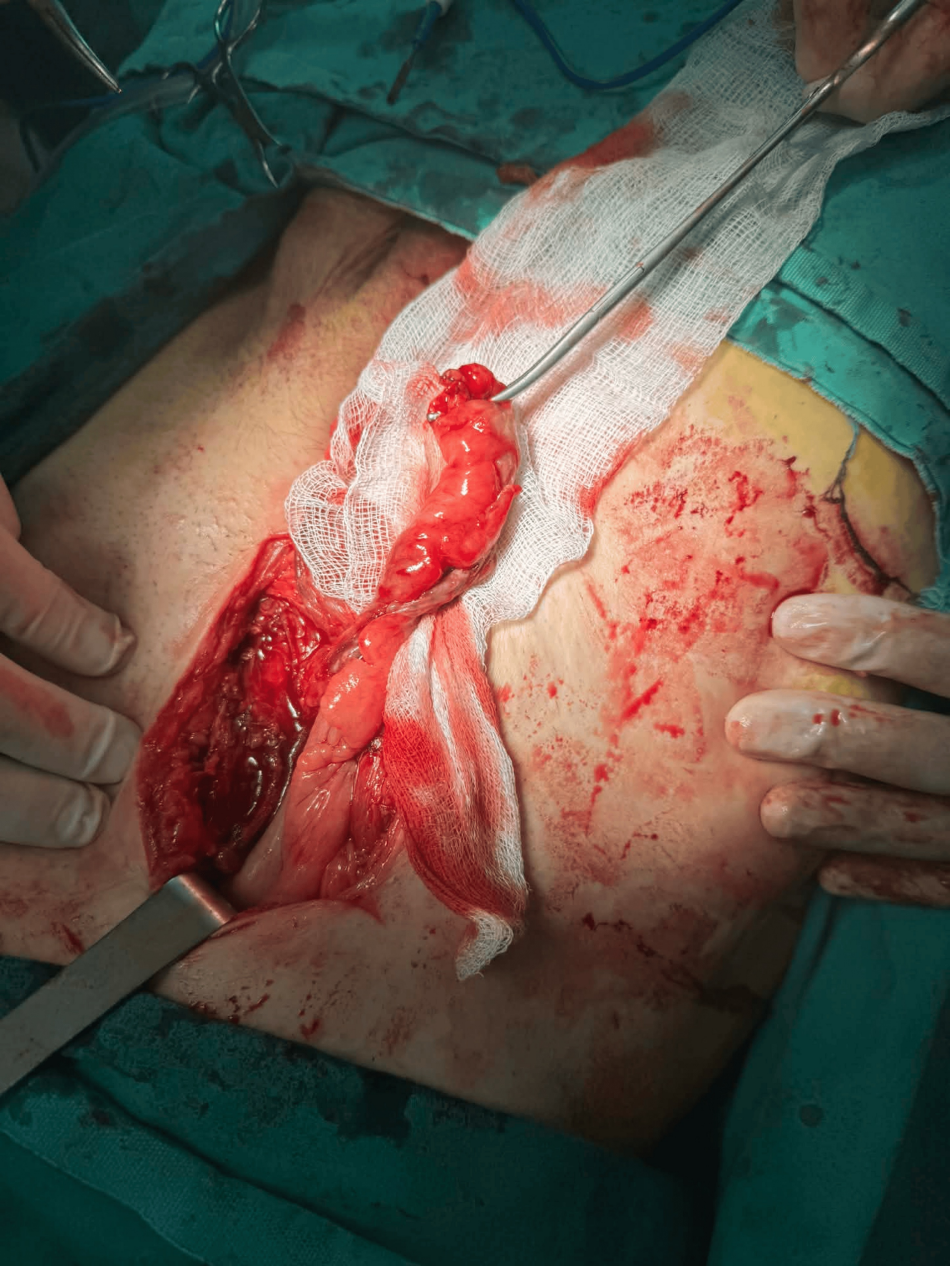
Operative field after appendectomy and peritoneal lavage with preserved mesh Post-appendectomy view demonstrating a clean surgical field following extensive lavage. The previously implanted mesh was preserved as it was not involved in the inflammatory process

The final operative field after appendectomy and lavage, with the preservation of the previously implanted mesh, is shown (Figure 4).

The microbiological analysis of peritoneal fluid revealed *Escherichia coli* sensitive to piperacillin/tazobactam and other broad-spectrum antibiotics.

The patient received intravenous piperacillin/tazobactam (4.5 g three times daily) and metronidazole (500 mg three times daily) for five days.

The postoperative course was uneventful. No fever was recorded, and inflammatory markers gradually declined, with the normalization of leukocyte count.

At one-month follow-up, the patient was asymptomatic. Clinical examination confirmed complete wound healing with no evidence of infection or hernia recurrence. Long-term follow-up is required to fully assess recurrence risk.

## Discussion

Amyand’s hernia represents a rare and often unexpected intraoperative finding [3].

According to the Losanoff and Basson classification, this case corresponds to types II-III due to the presence of an inflamed appendix and local contamination [1].

The optimal surgical approach remains debated. In cases complicated by inflammation or contamination, appendectomy combined with primary tissue repair without mesh is generally recommended [4,5].

The use of prosthetic mesh in contaminated fields is associated with an increased risk of surgical site infection and prosthetic complications [6]. However, the management of previously implanted mesh is less clearly defined. Previous reports of Amyand’s hernia have described different operative strategies depending on the degree of appendiceal inflammation and contamination [1-5,7-10]. In uncomplicated cases, mesh repair may be safely performed, whereas inflamed or perforated cases are generally managed with appendectomy and the avoidance of new prosthetic material. Compared with previously reported cases, the distinctive feature of the present case was the presence of previously implanted mesh within a contaminated operative field. This created a more complex intraoperative decision-making process, as the risk of mesh infection had to be balanced against the morbidity associated with mesh removal.

In the present case, the preservation of the existing mesh was chosen, as it was not directly involved in the inflammatory process.

The rationale for preserving the previously implanted mesh was based on the absence of direct prosthetic involvement and the lack of visible infection surrounding the mesh. In addition, preservation avoided additional tissue trauma and prolonged operative dissection. The favorable postoperative outcome observed in this patient supports the importance of individualized surgical assessment in complex contaminated Amyand’s hernia cases.

Microbiological findings confirmed *Escherichia coli*, a common pathogen in intra-abdominal infections, supporting the use of broad-spectrum antimicrobial therapy [7].

The favorable postoperative course and absence of recurrence at one-month follow-up support the safety and effectiveness of this approach.

## Conclusions

This case demonstrates that a contaminated Amyand’s hernia in a previously mesh-repaired groin requires the careful intraoperative assessment of both the appendix and the existing prosthetic material. In this patient, the preservation of the previously implanted mesh was associated with an uncomplicated postoperative recovery because no direct prosthetic involvement was identified intraoperatively.
